# Take a Bite! The Effect of Bitten Food in Pictures on Product Attitudes, Purchase Intentions, and Willingness to Pay

**DOI:** 10.3390/foods10092096

**Published:** 2021-09-04

**Authors:** Eva Meersseman, Maggie Geuens, Iris Vermeir

**Affiliations:** Department of Marketing, Innovation and Organisation, Faculty of Economics and Business Administration, Ghent University, 9000 Ghent, Belgium; maggie.geuens@ugent.be (M.G.); iris.vermeir@ugent.be (I.V.)

**Keywords:** food pictures, visual elements, design, purchase intentions, willingness to pay, product attitudes, disgust, consumer contamination, embodied mental simulation

## Abstract

Food pictures in advertisements, on packages, and on social media often display food with a bite in it. We investigated the effect of pictures of food with a bite (vs. no bite) on product attitudes, purchase intentions, and willingness to pay. In two online experimental studies we tested this effect for both pictures without context, as well as pictures in an advertisement. We also investigated two theories that could lead to opposite effects: consumer contamination and embodied mental simulation. We found that a picture of food with a bite (vs. no bite) resulted in lower purchase intentions, and that this effect was mediated by disgust (i.e., consumer contamination). Furthermore, we found an interaction effect of picture type (i.e., bite vs. no bite) and context (i.e., no context vs. advertisement) on purchase intentions: the effect of picture type on purchase intentions was attenuated when the picture appeared in an advertisement (vs. when the picture is shown without context). We found similar effects on product attitudes and willingness to pay. Lastly, a picture of food with a bite (vs. no bite) had no effect on embodied mental simulation. Field practitioners are advised to take caution when using pictures of bitten food as this may lead to unfavorable consumer responses because of a feeling if disgust.

## 1. Introduction

Food pictures are omnipresent on social and commercial media. Indeed, consumers are exposed to food pictures on a daily basis: from scrolling through a food-themed Instagram account to driving past a billboard promoting a new hamburger; from ordering a meal on a delivery site to flicking through a grocery store magazine with food advertisements [1,2,3].

Previous research has already shown the importance of visual elements, and more specifically how food is presented in food pictures, in guiding consumer food-related behavior [4]. For example, the camera angle in food pictures has been found to affect consumers’ food choices. When consumers were exposed to food pictures using a top perspective (i.e., photographing the food from above) instead of a diner’s eye perspective (i.e., mimicking the viewing point of a person sitting at a table looking at food on the table), they chose less unhealthy food options [5]. Furthermore, another research showed that consumers underestimate the calorie content of a burger in a picture when it is accompanied with a healthy side (i.e., celery sticks), compared to when the burger is pictured alone [6].

In the current research, we look at the unexplored effect of another popular product presentation element within food pictures on consumers’ product attitudes, purchase intentions, and willingness to pay. Specifically, we compare consumer reactions to pictures of food pictures of food displayed as partly eaten (i.e., a bite having been taken of the food) with pictures of the same food displayed as untouched (i.e., no part has yet been eaten of the food).

Pictures of food out of which a bite has been taken are popular in advertisements, on product packages, and on social media platforms. Sometimes, the bite in the food serves a specific purpose: to expose the inside of the food (e.g., a jam-filled donut, a chocolate coated biscuit, …) Yet, often, the bite in the food gives no extra information about the food product compared to a picture of the same non-bitten food. In the latter case, it is thus purely a stylistic choice. Based on previous consumer behavior research, we expect that this visual element in food pictures will have an effect on consumer responses (e.g., product attitudes, purchase intentions, and willingness to pay). In the next two sections, we will elaborate on two rival theories that would predict opposite consumer reactions to pictures displaying food with (vs. without) a bite.

### 1.1. Theory of Consumer Contamination

Consumers have a tendency to touch products in a store, in order get a better idea of the material and other tactical aspects. Nevertheless, when deciding to buy the product, they tend to take another ‘fresh’ and ‘untouched’ exemplar of the same product, although there is objectively nothing inferior about the touched product. Furthermore, consumers are less inclined to buy a product that they believe has been touched by other consumers. This phenomenon is referred to as consumer contamination [7]. Research showed that consumers have more negative reactions to products when there are cues that indicate they have been touched by other consumers. For example, used clothing is often rejected by consumers because of a fear of contamination [8]. Argo et al. [7] set up an experiment in which participants were exposed to a realistic shopping experience, and were invited to try on a t-shirt. In three different conditions, participants were led to believe there was only one t-shirt left in the store, but either (a) someone was trying this t-shirt on at the moment; (b) the t-shirt was hanging on the return rack; or (c) the t-shirt was hanging on its allocated rack. Participants that were in the contamination conditions (a and b) indicated more negative product attitudes towards the t-shirt (although the t-shirt had not actually been tried on, so it was exactly the same in every condition). Disgust is found to be the mediating variable driving this effect. Furthermore, the researchers discovered that the more salient the cue of ‘contamination’ is (i.e., in condition (a) the cue was most salient, as another person was trying on the t-shirt right at that moment), the stronger the effect is [7].

Consumers can also experience a feeling of disgust towards food that has been touched by other people. Rozin ([9], p. 23) defines food-related disgust as “revulsion at the prospect of (oral) incorporation of an offensive object.” A contaminant makes the food that it touches less acceptable (e.g., an unknown person touching a food product in store) [9]. Even when the source of disgust is removed from the food item (e.g., the other person is not touching the food anymore; the fly is removed from the soup) consumers still fear that there are traces of the source of disgust on the food [10]. Furthermore, merely suspecting that a source of disgust has been in contact with the food is enough for the consumers to reject the food product [11].

The theory of consumer contamination is relevant to the current research, as we used pictures of bitten food. Even though it is obvious that the consumer will not be presented to buy a product that has partly been consumed by someone else, it is conceivable that pictures of bitten food suggest that someone else has touched the food and thus automatically evoke perceptions of consumer contamination. The bite could therefore elicit a feeling of disgust. Hence, we propose the following:

**Hypothesis** **1a** **(H1a).** *A picture of food with a bite (vs. no bite) reduces product attitudes, purchase intentions, and willingness to pay.*

**Hypothesis** **1b** **(H1b).** *The “bite effect” is driven by an enhanced feeling of disgust.*

Nevertheless, we expect that the effect is dependent on the context the picture of the food with a bite (vs. no bite) appears in. We argue that consumers could react differently to pictures of bitten food if this picture is shown as part of an advertisement or when the picture of the bitten food is presented in the absence of any other information (like, for example, a picture of a bitten food item at a canteen counter or a supermarket display). We argue that the disgust people experience when seeing a bitten food item in an advertisement could be countered, since the context of the advertisement makes consumers realize that they would not receive the actual bitten food in the picture; the displayed food item is after all only a communication image, rather than a picture of the real product they would consume. Consumers know that when they decide to buy a product based on an ad they will not be receiving the actual product in the ad picture, but another ‘untouched’ product. We propose the following:

**Hypothesis** **1c** **(H1c).** *The “bite effect” is moderated by context in the sense that the effect of a picture of food with (vs. without) a bite is less pronounced when the picture is displayed in an advertisement (vs. when it is shown without context).*

### 1.2. Theory of Embodied Mental Simulation

The theory of embodied mental simulation would predict the opposite results of pictures of food with a bite (vs. no bite) on consumer responses.

Embodied mental simulation is an automatic tendency of people to imagine interacting with an object (e.g., eating a food product) that is elicited by a verbal or visual representation of the object (e.g., reading a slogan about the product, looking at a picture of the product) [12]. Research revealed that the way a product is visually presented can affect the intensity with which consumers mentally interact with the product. For example, when looking at pictures of food shot with a diner’s eye camera angle (vs. a vertically downward camera angle), the mental simulation of eating the food was more intense (i.e., participants could, to a higher extent, imagine eating the food) [5]. Furthermore, when a static picture implies motion (e.g., a picture of water being poured into a glass), people will imagine the motion as if it were happening at that moment, activating similar areas of the brain as if they were seeing the actual motion [13,14,15].

Furthermore, research found that when it is easier to imagine consuming food (i.e., higher product vividness) and/or easier to imagine sensory aspects (i.e., the taste, smell, and texture) of a product, desire for the product and/or the need for instant gratification (i.e., the need to immediately satisfy your cravings for a product) are higher [5,16,17,18,19].

Lastly, research found that when exposing participants to pictures of a model in different stages of eating the food (e.g., before, during, and after eating a spoon of yoghurt), the sensory imagery of eating the yoghurt and the desire for the yoghurt was highest when seeing the picture of the model during consumption [18]. In our study, we used a picture of food with a bite, which could be interpreted as a picture taken during consumption.

Hence, based on the theory of embodied mental simulation and the studies mentioned above, we could argue that (in contrast to H1a), pictures of food with a bite (vs. no bite) would generate higher product attitudes, purchase intentions, and a willingness to pay. If a picture displays food with a bite (vs. no bite), consumers could experience a greater ease in imagining consuming this food and in imagining the sensory aspects of the food. Therefore, participants’ desire for the food and need for instant gratification by the food would be higher. This could result in more positive consumer responses to the food. In sum, we formulate the following rival hypotheses to H1a and H1b:

**Hypothesis** **2a** **(H2a).** *A picture of food with a bite (vs. no bite) increases product attitudes, purchase intentions, and willingness to pay.*

**Hypothesis** **2b** **(H2b).** *The “bite effect” is driven by enhanced embodied mental simulation.*

In conclusion, we consider two theories that would predict opposite effects of a picture with (vs. without) a bite on consumer responses. More specifically, the theory of consumer contamination predicts a negative effect, while the theory of embodied mental simulation predicts a positive effect on consumer responses. Therefore, we will test the effect of picture type on consumer responses, taking both theories into account as possible mediators of the main effect.

## 2. Study 1

### 2.1. Study Overview

In this study, we tested whether a picture of food with a bite (vs. without a bite) lead to lower (H1a) or higher (H2a) product attitudes, purchase intentions, and willingness to pay. Furthermore, we tested whether a possible main effect was mediated by an increased feeling of disgust (H1b) or an increased embodied mental simulation (H2b).

### 2.2. Participants, Stimuli, and Procedure

**Figure 1:**
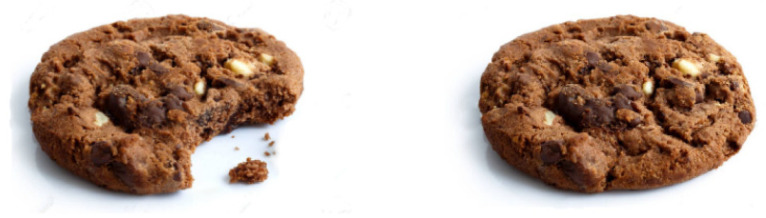
The used stimuli for Study 1.

For study 1, we recruited 121 British participants on Prolific (41% male, M_age_ (mean age) = 36.15, SD_age_ (standard deviation of age) = 14.84). Study 1 was a between-subjects two-conditions study. Participants saw either a picture of a cookie with a bite or no bite taken out of it (see Table A1 of the Appendix A for demographics of the different conditions). The cookie was neither filled, nor was it coated. Hence, the bite in the cookie provided no additional information about the characteristics of the cookie. Figure 1 shows the pictures used in Study 1.

Participants were randomly and evenly assigned to one of the two conditions and were exposed to the corresponding stimulus. First they saw the cookie, then participants’ product attitudes, purchase intentions, and willingness to pay for the cookie were measured in a random order. Product attitudes were measured on a ten item 7-point Likert scale (“I think this product is unattractive: attractive; low quality: high quality; bad: good; negative: positive; unenjoyable: enjoyable; not tasty: tasty; undesirable: desirable; unfavorable: favorable; dislikeable: likeable; unhealthy: healthy”). After controlling for scale reliability with Cronbach’s alpha (α = 0.68), the items were summated and averaged. This average was to be used in the analyses. The same procedure was used for other multi-item measures throughout this manuscript. Purchase intentions were measured by means of three items on a 7-point bipolar scale (“How likely would you be to buy this product? Very unlikely to buy this product: Very likely to buy this product; Very unwilling to buy this product: Very willing to buy this product; Very uninclined to buy this product: Very inclined to buy this product”) (α = 0.75) [20]. Willingness to pay was measured on a scale ranging from −2 to 2 Pounds (£) with intervals of £0.5. Participants were asked: “How much more or less are you willing to pay for a pack of 12 of these cookies compared to a pack of a similar brand?”.

Next, participants’ disgust towards the cookie, product vividness, sensory imagery, desire, and need for instant gratification were measured in a random order. We created three items to measure disgust on a 7-point bipolar scale. The items are: “The way this biscuit is presented somehow attracts me: somehow repels me”; “The way this biscuit is presented gives me the impression that it is fresh: not fresh anymore”; and “The way this biscuit is presented, makes me somewhat feel that the biscuit is hygienic and not contaminated: somewhat unhygienic and contaminated” (α = 0.71). We reformulated items from the scale used by Argo et al. (2006) to measure the disgust consumers were feeling during a shopping experience when using an actual product that they believed had been previously touched by other consumers. Instead of asking whether participants felt “revolted”, or “gross”), we used less extreme measures of disgust (i.e., “somewhat repelled me”) since it is unlikely that consumers experience those intense emotions in the context of food that is not spoiled or contaminated.

In order to measure product vividness and sensory imagery, participants were asked to look at the picture of the cookie for 20 s. They were instructed to imagine eating the cookie while looking at it. Next, participants filled out a 7-point bipolar scale with three items measuring product vividness (“While looking at the picture, I found it …” “not easy: easy to visualize myself consuming the biscuit”; “not easy: easy to imagine myself consuming the biscuit”; “not easy: easy to picture myself consuming the biscuit”) (α = 0.81) [16] and a 7-point Likert scale with three items measuring sensory imagery (“While looking at the picture… I could imagine the taste of the biscuit”, “I could imagine the smell of the biscuit”, “I could imagine the texture of the biscuit”) (α = 0.74) [18]. Desire was measured with three items on a 7-point Likert scale (“When viewing the picture, I experience the desire to eat this biscuit”, “I would like to eat this biscuit after the study ends”, “If I had this biscuit at hand, I would immediately eat it”) (α = 0.89) [18]. Lastly, participants’ need for instant gratification was measured with a four-item bipolar scale (“While indicating to what extent I wanted to buy the biscuit, my goal (unconsciously or not) was one of …” “avoiding gratification: seeking gratification”, “avoiding pleasure: seeking pleasure”, “keeping my impulses in check: satisfying my impulses”, and “avoiding indulging: indulging”) (α = 0.91) [17].

Finally, participants indicated their gender and age, added comments if wanted, were thanked for their participation, and were dismissed.

### 2.3. Statistical Analysis

We performed an independent samples t-test with picture type (i.e., bite vs. no bite) as the independent variable, and product attitudes, purchase intentions, willingness to pay, disgust, vividness, sensory imagery, desire and need for instant gratification as the dependent variables. We also performed three simple mediation analyses [21] with picture type (i.e., bite vs. no bite) as the dependent variable, disgust as the mediator, and product attitudes, purchase intentions and willingness to pay as the dependent variables. All analyses were performed with the statistical program SPSS (Statistical Package for the Social Sciences).

### 2.4. Results

#### 2.4.1. Main Effect on Product Attitudes, Purchase Intentions, and Willingness to Pay

Product attitudes. Lastly, product attitudes significantly differed among participants exposed to the picture of a cookie with a bite versus no bite, t(102.26) = 2.32, *p* = 0.022. The picture of the bitten cookie generated lower product attitudes (M = 5.21, SD = 0.83) than the picture of the cookie that was not bitten (M = 5.49, SD = 0.48).

Purchase intentions. There was a difference in purchase intentions between both conditions, t(104.2) = 2.91, *p* = 0.004. Participants that saw a picture of a cookie with a bite taken out of it, had lower purchase intentions (M = 5.07, SD = 1.31) compared to participants that saw a picture with a complete cookie (M = 5.64, SD = 0.78).

Willingness to pay. Next, no difference in willingness to pay was detected between participants that saw a cookie pictured with a bite (M = 0.32, SD = 0.66) versus no bite (M = 0.25, SD = 0.82), t(119) = 2.91, *p* = 0.542.

#### 2.4.2. Consumer Contamination

Disgust was significantly higher for the picture with the bitten cookie (M = 3.41, SD = 1.17) compared to the cookie without a bite taken out of it (M = 2.96, SD = 1.14), as indicated by an independent samples t-test, t(119) = 2.16, *p* = 0.033.

**Figure 2:**
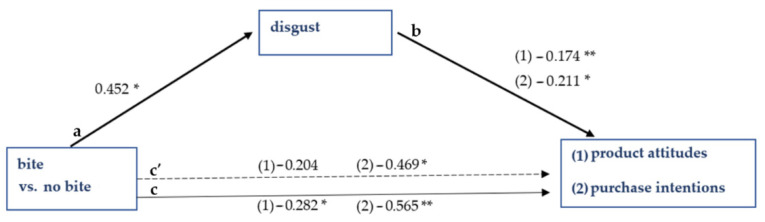
Results of Study 1. The effect of picture type on product attitudes and purchase intentions is mediated by disgust. Note: ** significant at *p* < 0.01; * significant at *p* < 0.05; a = effect of independent variable on mediating variable; b = effect of mediating variable on dependent variable; c´ = direct effect of independent variable on dependent variable; c = total effect of independent variable on dependent variable; (1) product attitudes; (2) purchase intentions.

Furthermore, disgust is a significant mediator of the effect of the cookie picture with a bite versus no bite on product attitudes (ab (indirect effect of independent on dependent variable) = −0.079, 95% CI (confidence interval) = −0.119 to −0.014) and purchase intentions (ab = −0.096, 95% CI = −0.201 to −0.007). The mediation is partial for purchase intentions, and full for product attitudes. Figure 2 shows the schematic overview of both mediation analyses. Lastly, disgust is no significant mediator of the effect of picture type on willingness to pay (ab = −0.012, 95% CI = −0.086 to 0.044).

#### 2.4.3. Embodied Mental Simulation

An independent samples t-test showed no differences between the picture of a cookie with a bite versus no bite taken out of it for vividness (t(97, 56) = 0.71, *p* = 0.477; M_bite_ = 5.67, SD_bite_ = 1.60; M_nobite_ = 5.83, SD_nobite_ = 1.84), the intensity of sensory imagery (t(119) = 0.27, *p* = 0.789; M_bite_ = 5.29, SD_bite_ = 1.10; M_nobite_ = 5.24, SD_nobite_ = 1.06), desire (t(119) = 0.71, *p* = 0.480; M_bite_ = 4.92, SD_bite_ = 1.15; M_nobite_ = 5.11, SD_nobite_ = 1.03), and participants’ need for instant gratification (t(119) = 0.388, *p* = 0.699; M_bite_ = 5.56, SD_bite_ = 1.03; M_nobite_ = 5.64, SD_nobite_ = 1.22).

## 3. Study 2

### 3.1. Study Overview

In this study, we firstly aimed to replicate the results of Study 1. Thus, we tested whether a picture of food with a bite (vs. without a bite) lead to lower (H1a) or higher (H2a) product attitudes, purchase intentions, and willingness to pay. Furthermore, we again tested whether the main effect was mediated by an increased feeling of disgust (H1b) or an increased embodied mental simulation (H2b). Furthermore, we tested whether the “bite effect” was more pronounced when the picture was shown in an advertisement (vs. when it was shown without a context) (H1c).

### 3.2. Participants, Stimuli, and Procedure

**Figure 3:**
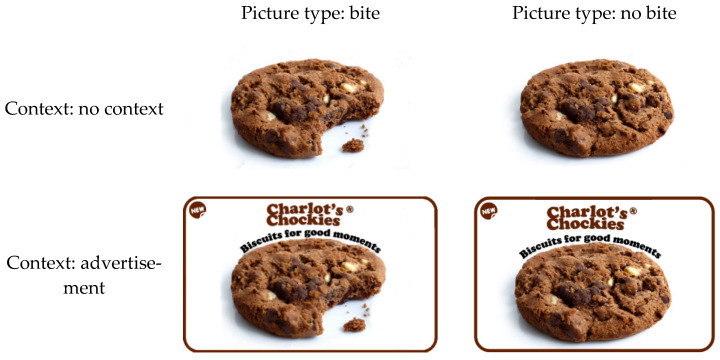
The used stimuli for Study 2.

For study 2, we recruited 272 British participants on Prolific (33,5% male, M_age_ = 34.72, SD_age_ = 13.02). Study 2 was a between-subjects study with a 2 (picture type: bite, no bite) x 2 (context: no context, advertisement) design (see Table A2 of the Appendix A for demographics of the different conditions). Seven participants were excluded from analysis, as they mentioned that they were allergic to chocolate, did not like chocolate, or did not like chocolate biscuits. Figure 3 shows the pictures used in Study 2.

The procedure in Study 2 was exactly the same as in Study 1. Participants were randomly and evenly assigned to one of the four conditions and were exposed to the corresponding stimulus. First they saw the cookie, then participants’ product attitudes (α = 0.92), purchase intentions (α = 0.95), and willingness to pay for the cookie were measured in a random order. Next, participants’ disgust towards the cookie (α = 0.72), product vividness (α = 0.95), sensory imagery (α = 0.73), desire (α = 0.92), and need for instant gratification (α = 0.90) were measured in a random order. Lastly, participants indicated their gender and age, added comments if wanted, were thanked for their participation, and were dismissed.

### 3.3. Statistical Analysis

We performed eight 2 × 2 ANOVA (analysis of variance) taking picture type (bite vs. no bite) and context (advertisement vs. no context) as independent variables and product attitudes, purchase intentions, willingness to pay, disgust, vividness, sensory imagery, desire, and need for instant gratification as dependent variables. In the 2 × 2 ANOVA analyses, we investigated the main effects of picture type, the main effects of context, and interaction effects of picture type and context. We also performed several independent samples t-tests to compare the responses of respondents seeing an advertisement with a picture of food with a bite or without a bite, and to compare the responses of respondents seeing a picture without context of the food with a bite or without a bite. We also performed three simple mediation analyses [21] with picture type (i.e., bite vs. no bite) as the dependent variable, disgust as the mediator, and product attitudes, purchase intentions, and willingness to pay as the dependent variables. All analyses were performed with the statistical program SPSS.

### 3.4. Results

#### 3.4.1. Main Effects on Purchase Intentions, Willingness to Pay, and Product Attitudes

**Figure 4:**
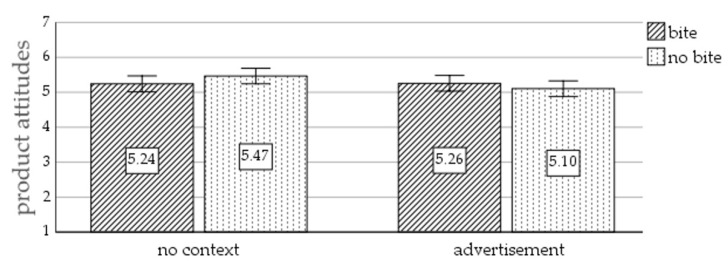
Results of Study 2. The interaction of picture type and context on product attitudes. Note: error bars represent 95% confidence interval.

Product attitudes. There was no main effect of picture type on product attitudes, F(1, 268) = 0.094, *p* = 0.760, η² = 0.000 (M_bite_ = 5.25, SD_bite_ = 0.95; M_nobite_ = 5.28, SD_nobite_ = 0.94). Furthermore, there was no main effect of context on product attitudes, F(1, 268) = 2.34, *p* = 0.128, η² = 0.009 (M_nocontext_ = 5.36, SD_nocontext_ = 0.90; M_ad_ = 5.18, SD_ad_ = _0_.98). Next, a marginally significant interaction effect of picture type and context on product attitudes was revealed, F(1, 268) = 2.75, *p* = 0.098, η² = 0.010. Figure 4 displays the product attitudes for the four conditions. Product attitudes did not significantly differ for participants exposed to the picture without context of the cookie with a bite (M = 5.24, SD = 0.92) versus the whole cookie (M = 5.47, SD = 0.88), t(1, 133) = 1.45, *p* = 0.150). No significant difference in product attitudes was detected for participants that saw an advertisement containing a picture of a cookie with a bite (M = 5.26, SD = 0.99) or no bite (M = 5.10, SD = 0.97), t(1, 135) = 0.92, *p* = 0.358. 

**Figure 5:**
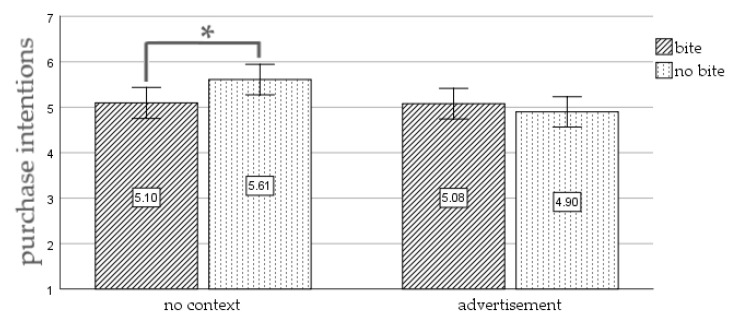
Results of Study 2. The interaction of picture type and context on purchase intentions. Note: * significant at *p* < 0.05; error bars represent 95% confidence interval.

Purchase intentions. We found no significant main effect of picture type (i.e., a picture with a bitten cookie versus a cookie without a bite) on purchase intentions, F(1, 268) = 0.95, *p* = 0.331, η² = 0.004 (M_bite_ = 5.09, SD_bite_ = 1.46; M_nobite_ = 5.25, SD_nobite_ = 1.39). Next, a significant main effect of context (i.e., a picture without context versus a picture in an advertisement) on purchase was detected, F(1, 268) = 4.54, *p* = 0.034, η² = 0.017) (M_nocontext_ = 5.36, SD_nocontext_ = 1.35; M_ad_ = 4.99, SD_ad_ = 1.48). Furthermore, the analysis revealed a significant interaction effect of picture type and context on purchase intentions, F(1, 268) = 4.11, *p* = 0.044, η² = 0.015. Figure 5 displays the purchase intentions of the four conditions. Purchase intentions of participants exposed to the picture without context were significantly lower for the picture of the cookie with a bite (M = 5.10, SD = 1.47) versus a picture of the complete cookie (M = 5.61, SD = 1.19), t(1, 133) = 2.24, *p* = 0.027. No significant difference in purchase intention was detected for participants that saw an advertisement with a picture of a cookie with a bite (M = 5.08, SD = 1.47) or no bite (M = 4.90, SD = 0.97), t(1, 135) = 0.77, *p* = 0.487.

**Figure 6:**
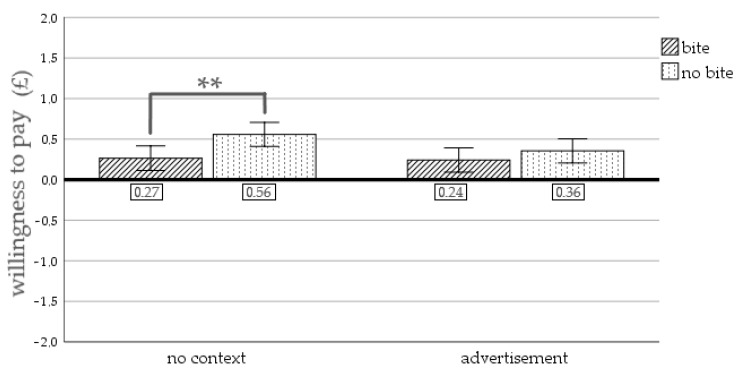
Results of Study 2. The interaction of picture type and context on willingness to pay. Note: ** significant at *p* < 0.01; error bars represent 95% confidence interval.

Willingness to pay. A significant main effect of picture type on willingness to pay was revealed, F(1, 268) = 7.08, *p* = 0.008, η² = 0.026 (M_bite_ = 0.25, SD_bite_ = 0.63; M_nobite_ = 0.46, SD_nobite_ = 0.63). There was no significant main effect of context on willingness to pay, F(1, 268) = 2.19, *p* = 0.140, η² = 0.008 (M_nocontext_ = 0.41, SD_nocontext_ = 0.59; M_ad_ = 0.30, SD_ad_ = 0.68). The analysis revealed no significant interaction effect of picture type and context on willingness to pay, F(1, 268) = 1.40, *p* = 0.237, η² = 0.005. Figure 6 displays the willingness to pay for the different conditions. Willingness to pay of participants seeing a picture without context was significantly lower when the pictured cookie was bitten (M = 0.27, SD = 0.58) versus when there was no bite taken out of it (M = 0.56, SD = 0.56), t(1, 132.01) = 2.98, *p* = 0.003. No significant difference in willingness to pay was detected for participants that saw an advertisement containing a picture of a cookie with a bite (M = 0.24, SD = 0.68) or no bite (M = 0.36, SD = 0.68), t(1, 135) = 0.97, *p* = 0.335.

#### 3.4.2. Consumer Contamination

We found a significant main effect of picture type on disgust, F(1, 268) = 4.35, *p* = 0.038, η² = 0.016. Disgust was higher when the cookie in the picture was bitten (M = 3.36, SD = 1.68) versus when there was no bite taken out of it (M = 3.00, SD = 1.25). There was no significant main effect of context on disgust, F(1, 268) = 2.72, *p* = 0.100, η² = 0.010 (M_nocontext_ = 3.32, SD_nocontext_ = 1.70; M_ad_ = 3.04, SD_ad_ = 1.23). Finally, there was a significant interaction effect of picture type and context on disgust, F(1, 268) = 6,65, *p* = 0.010, η² = 0.024. Disgust of participants seeing a picture without context was significantly higher when the pictured cookie was bitten (M = 3.74, SD = 1.95) versus when there was no bite taken out of it (M = 2.92, SD = 1.03), t(1, 112.59) = 2.87, *p* = 0.005. No significant difference in disgust was detected for participants that saw an advertisement with a picture of a cookie with a bite (M = 3.00, SD = 1.26) or no bite (M = 3.08, SD = 1.20), t(1, 135) = 0.41, *p* = 0.680.

**Figure 7:**
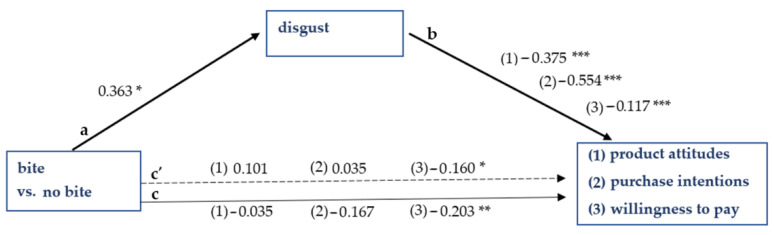
Results of Study 2. The effect of picture type on product attitudes, purchase intentions, and willingness to pay is mediated by disgust. Note: *** significant at *p* < 0.001; ** significant at *p* < 0.01; * significant at *p* < 0.05; a = effect of independent variable on mediating variable; b = effect of mediating variable on dependent variable; c’ = direct effect of independent variable on dependent variable; c = total effect of independent variable on dependent variable.

Furthermore, disgust is a significant mediator of the effect of picture type on product attitudes (ab = −0.136, 95% CI = −0.276 to −0.005), purchase intentions (ab = −0.201, 95% CI = −0.413 to −0.012), and willingness to pay (ab = −0.043, 95% CI = −0.102 to −0.005), regardless of the context the picture appears in. The mediation is full for purchase intentions and product attitudes, and partial for willingness to pay. Figure 7 gives a schematic overview of the three mediation analyses.

#### 3.4.3. Embodied Mental Simulation

Analysis revealed no main effects of picture type on vividness (F(1, 268) = 0.05, *p* = 0.834, η² = 0.000), sensory imagery (F(1, 268) = 0.42, *p* = 0.515, η² = 0.002), desire (F(1, 268) = 0.01, *p* = 0.910, η² = 0.000), and need for instant gratification (F(1, 268) = 1.04, *p* = 0.308, η² = 0.004). We found a marginally significant main effect of context on vividness, F(1, 268) = 2.99, *p* = 0.085, η² = 0.011 (M_nocontext_ = 6.04, SD_nocontext_ = 1.07; M_ad_ = 5.78, SD_ad_ = 1.37) and a significant main effect of context on desire, (F(1, 268) = 6.04, *p* = 0.015, η² = 0.022 (M_nocontext_ = 5.44, SD_nocontext_ = 1.33; M_ad_ = 5.00, SD_ad_ = 1.59). There was no main effect of context on sensory imagery (F(1, 268) = 0.67, *p* = 0.415, η² = 0.002) and need for instant gratification (F(1, 268) = 0.36, *p* = 551, η² = 0.001). Lastly, no interaction effects of picture type and context were detected on vividness (F(1, 268) = 0.14, *p* = 0.707, η² = 0.001), sensory imagery (F(1, 268) = 0.06, *p* = 0.813, η² = 0.000), desire (F(1, 268) = 0.17, *p* = 0.679, η² = 0.001), and need for instant gratification (F(1, 268) = 0.09, *p* = 0.764, η² = 0.000).

## 4. General Discussion

### 4.1. Summary of Findings

Our findings provide full support to H1a, H1b, and H1c for the dependent variable purchase intention, and partial support for willingness to pay and product attitudes. Hence, we find no evidence for the rival hypotheses H2a and H2b.

Study 1 showed significantly higher product attitudes and purchase intentions for a picture of food with a bite (vs. no bite). Both effects are mediated by an increased feeling of disgust. This is in line with the theory of consumer contamination [7,8,9,10,11], which shows that consumers have more negative reactions to products when they have been touched or contaminated by another person or another source of disgust. However, willingness to pay was not affected by picture type, and disgust did not mediate the effect of picture type on willingness to pay. Perhaps, a picture with (vs. without) a bite only has a significant effect on attitudes and intentions (i.e., product attitude and purchase intention), but not on behavior (i.e., willingness to pay). This is not in line with the theory of planned behavior, which shows that attitudes are closely related to behavior [22]. However, research has also showed that attitude strength has an effect on behavior [23,24,25]. Possibly, the attitudes towards the pictures were not very strong, which resulted in insignificant differences in willingness to pay. Lastly, a picture of food with a bite (vs. no bite) had no effect on product vividness, sensory imagery, desire, and need for instant gratification. Therefore, the theory of embodied mental simulation cannot explain the results. Perhaps, the depiction of food with (vs. without) a bite did not lead to a difference in vividness, as both types of pictures are already quite vivid, regardless of the bite.

Next, Study 2 revealed a significant interaction effect of the picture type (i.e., picture with bite vs. no bite) and context (i.e., no context vs. advertisement) on purchase intentions and a marginally significant interaction effect on product attitudes. Participants that were exposed to a picture without context indicated significantly lower purchase intentions when the food in the picture had a bite (vs. no bite) taken out of it. No significant interaction effect was found on willingness to pay. Nevertheless, participants that saw a picture without context had a significantly lower willingness to pay when the food in the picture had a bite (vs. no bite) taken out of it. Furthermore, there was a main effect of picture type on willingness to pay: participants were willing to pay less when the cookie in the picture had a bite (vs. no bite). So again, it seems that a picture with a bite (vs. no bite) mostly has an effect on consumers’ attitudes and intentions (i.e., product attitude and purchase intentions), and to a lesser extent on behavior (willingness to pay). The effect of a picture with a bite (vs. no bite) on purchase intentions, product attitudes and willingness to pay was mediated by disgust. Therefore, Study 2 also confirms the consumer contamination theory [7,8,9,10,11]. No main effects of picture type, and interaction effect of picture type and context, were detected on product vividness, sensory imagery, desire, and need for instant gratification. This confirms again the rejection of the theory of embodied mental simulation as an explanation for the effects found.

### 4.2. Theoretical and Managerial Implications

The current work makes three theoretical contributions. First, it adds to the literature of food pictures and its effect on consumer behavior. We add a visual element in food pictures that can influence product attitudes, purchase intentions, and willingness to pay: namely pictures of food with a bite vs. no bite. This popular visual element in food pictures in different contexts (e.g., advertisements, product packages, social media content, …) had been left unexplored until now.

Second, the research adds to the literature of consumer contamination. In addition to research showing the effects of contamination in a real-life context [7], we show that disgust can also be evoked by displaying pictures of “normal-looking” food products merely by suggesting that somebody took a bite from this product.

Third, the research adds to the literature of embodied mental simulation. Namely, it rules out a possible effect of pictures of food with a bite vs. no bite on embodied mental simulation. A bite in food does not make it easier for consumers to imagine consuming the food in the picture, nor imagining the sensory aspects of the food product. Therefore it does not lead to increased desire or the need for instant gratification.

Moreover, the current findings hold several implications for field practitioners in the food industry. The findings can help marketeers to decide on the right food picture to use in an advertisement, product packaging, etc. The research suggests marketers to be cautious when it comes to using pictures of food with a bite taken out of it. Of course, when the bite serves the purpose of revealing the inside of a product (e.g., a jam-filled donut), marketeers might feel compelled to show the product with a bite. Nevertheless, an option is to show the inside without making it appear like a bite, by cutting a straight piece (out) of the product. Furthermore, the results also have implications for society. It helps consumers to be aware of the impact subtle differences in food pictures have on their choices.

### 4.3. Limitations and Future Research Directions

Further research is needed in order to further generalize the findings of the current paper. First, in the current studies, one product (a cookie) was used to test the effect. Future research could use different types of food products to exclude idiosyncratic effects.

Second, in the current research, consumers’ attitudes and intentions were measured, but not their behavior. In the future, it could be valuable to set up a (field) study where participants make actual food choices and consume food. Furthermore, the current studies only used one product. Future research could test the effects on multiple products.

Third, disgust sensitivity differs cross-culturally. For example, Asian consumers are found to have a higher disgust sensitivity than Caucasian consumers [26]. In the current work, we only investigated British respondents. Therefore, the effects found could be different (i.e., stronger or weaker) in other cultures.

Fourth, the current studies took place during the COVID-19 pandemic (April–May 2021). During a time where sanitizing and social distancing are more important than ever, consumers’ disgust sensitivity may be stronger than before. Therefore, a picture of food that is bitten might elicit stronger feelings of disgust than what it did before the pandemic. Future research can replicate these studies after the pandemic.

Fifth, it could be interesting to test products with other consumption cues apart from a bite, such as a ‘sip’ or a ‘scoop’ (e.g., a drink of which a ‘sip’ has been taken and hence the bottle is not entirely full anymore, a cup of yoghurt or ice cream that shows a visible ‘scoop’ missing).

Sixth, future research could look into pictures of food with a bite (vs. a cut) to reveal the inside of the food (e.g., for a chocolate filled cookie). It would be interesting to see whether the negative effects of a bite on consumer reactions also hold there.

Finally, research could focus on other unexplored visual aspects in food images. For example, one could compare a picture of food to a drawing or animation of food. Namely, food on packages is often drawn (e.g., a drawing of an orange on a bottle of orange juice). Furthermore, a bite out of a food that is drawn might not lead to an increased feeling of disgust, as the product on the image is not real.

## 5. Conclusions

Pictures of bitten food (vs. complete food) diminish product attitudes, purchase intentions, and willingness to pay. This effect is mediated by a feeling of disgust (i.e., consumer contamination). Picture type does not affect mental simulation. There is an interaction effect of picture type (i.e., a picture of food with or without a bite) and context (a picture is shown in an advertisement or is shown without additional information) on purchase intentions: the effect of picture type on purchase intentions diminishes when the picture appears in an advertisement (vs. when it is shown without context).

## Data Availability

The data presented in this study are available on request from the corresponding author. Although consumer data have been anonymized, data are not publicly available.

## Appendix A

**Table A1:** Demographics of the different conditions in Study 1.

Condition	N	M_age_ (SD_age_)	% Men
Bite	64	37.86 (16.10)	45
No Bite	57	36.01 (13.14)	37

**Table A2:** Demographics of the different conditions in Study 2.

Condition	N	M_age_ (SD_age_)	% Men
Bite-Advertisement	69	35.42 (13.82)	33
No Bite-Advertisement	69	34.81 (12.89)	29
Bite-No Context	68	35.29 (13.33)	38
No Bite-No Context	66	33.29 (12.13)	33

## References

[1] Holmberg C., Chaplin J.E., Hillman T., Berg C. (2016). Adolescents’ presentation of food in social media: An explorative study. Appetite.

[2] Rousseau S. (2012). Food and Social Media: You are What you Tweet.

[3] Rousseau S., Thompson P.B., Kaplan D.M. (2014). Food “porn” in media. Encyclopedia of Food and Agricultural Ethics.

[4] Vermeir I., Roose G. (2020). Visual Design Cues Impacting Food Choice: A Review and Future Research Agenda. Foods.

[5] Meersseman E., Vermeir I., Geuens M. (2021). The effect of perspectives in food pictures on unhealthy food choices. Food Qual. Prefer..

[6] Chernev A. (2011). The Dieter’s Paradox. J. Consum. Psychol..

[7] Argo J.J., Dahl D.W., Morales A.C. (2006). Consumer Contamination: How Consumers React to Products Touched by Others. J. Mark.

[8] O’Reilly L., Rucker M., Hughes R., Gorang M., Hand S. (1984). The relationship of psychological and situational variables to usage of a second-order marketing system. J. Acad. Mark. Sci..

[9] Rozin P., Fallon A.E. (1987). A perspective on disgust. Psychol. Rev..

[10] Fallon A.E., Rozin P., Pliner P. (1984). The child’s conception of food: The development of food rejections with special reference to disgust and contamination sensitivity. Child Dev..

[11] Morales A.C., Fitzsimons G.J. (2007). Product Contagion: Changing Consumer Evaluations through Physical Contact with “Disgusting” Products. J. Mark. Res..

[12] Elder R.S., Krishna A. (2012). The “Visual Depiction Effect” in Advertising: Facilitating Embodied Mental Simulation through Product Orientation. J. Consum. Res..

[13] Mackert M., Lazard A., Guadagno M., Wagner H.J. (2014). The role of implied motion in engaging audiences for health promotion: Encouraging naps on a college campus. J. Am. Coll. Health.

[14] Blakemore S.J., Decety J. (2001). From the perception of action to the understanding of intention. Nat. Rev. Neurosci..

[15] Krekelberg B., Vatakis A., Kourtzi Z. (2005). Implied motion from form in the human visual cortex. J. Neurophysiol..

[16] Shiv B., Fedorikhin A. (1999). Heart and mind in conflict: The interplay of affect and cognition in consumer decision making. J. Cons. Res..

[17] Shiv B., Fedorikhin A. (2002). Spontaneous versus controlled influences of stimulus- based affect on choice behavior. Organ. Behav. Hum. Decis. Process..

[18] Palcu J., Haasova S., Florack A. (2019). Advertising models in the act of eating: How the depiction of different eating phases affects consumption desire and behavior. Appetite.

[19] Huyghe E., Verstraeten J., Geuens M., Van Kerckhove A. (2017). Clicks as a healthy alternative to bricks: How online grocery shopping reduces vice purchases. J. Mark. Res..

[20] White K., Lin L., Dahl D.W., Ritchie R.J. (2016). When do consumers avoid imperfections? Superficial packaging damage as a contamination cue. J. Mark. Res..

[21] Hayes A.F. (2017). Introduction to Mediation, Moderation, and Conditional Process Analysis: A Regression-Based Approac.

[22] Ajzen I. (1991). The theory of planned behavior. Organ. Behav. Hum. Decis. Process..

[23] Krosnick J.A., Boninger D.S., Chuang Y.C., Berent M.K., Carnot C.G. (1993). Attitude strength: One construct or many related constructs?. J. Personal. Soc. Psychol..

[24] Conner M., Sparks P., Povey R., James R., Shepherd R., Armitage C.J. (2002). Moderator effects of attitudinal ambivalence on attitude-behaviour relationships. Eur. J. Soc. Psychol..

[25] Kraus S.J. (1995). Attitudes and the prediction of behaviour—A meta-analysis of the empirical literature. Personal. Soc. Psychol. Bull..

[26] Haidt J., McCauley C., Rozin P. (1997). Individual differences in sensitivity to disgust: A scale sampling seven domains of disgust elicitors. Personal. Individ. Differ..

